# Localized mesospheric ozone destruction corresponding to isolated proton aurora coming from Earth’s radiation belt

**DOI:** 10.1038/s41598-022-20548-2

**Published:** 2022-10-11

**Authors:** Mitsunori Ozaki, Kazuo Shiokawa, Ryuho Kataoka, Martin Mlynczak, Larry Paxton, Martin Connors, Satoshi Yagitani, Shion Hashimoto, Yuichi Otsuka, Satoshi Nakahira, Ian Mann

**Affiliations:** 1 Graduate School of Natural Science and Technology, Kanazawa University, Kanazawa, Japan; 2 Institute for Space-Earth Environmental Research, Nagoya University, Nagoya, Japan; 3 National Institute of Polar Research, Tachikawa, Japan; 4 Department of Polar Science, The Graduate University for Advanced Studies, SOKENDAI, Tachikawa, Japan; 5 NASA Langley Research Center, Hampton, VA USA; 6 The Johns Hopkins University Applied Physics Laboratory, Laurel, MD USA; 7 Athabasca University Observatories, Athabasca, AB Canada; 8 Department of Physics and Astronomy, University of Calgary, Calgary, AB Canada; 9 Institute of Space and Astronautical Science, Japan Aerospace Exploration Agency, Sagamihara, Japan; 10 Department of Physics, University of Alberta, Edmonton, AB Canada

**Keywords:** Aurora, Magnetospheric physics

## Abstract

Relativistic electron precipitation (REP) from the Earth’s radiation belt plays an important role in mesospheric ozone loss as a connection between space weather and the climate system. However, the rapid (tens of minutes) destruction of mesospheric ozone directly caused by REP has remained poorly understood due to the difficulty of recognizing its location and duration. Here we show a compelling rapid correspondence between localized REP and ozone destruction during a specific auroral phenomenon, the called an isolated proton aurora (IPA). The IPA from the Earth’s radiation belt becomes an important spatial and temporal proxy of REP, distinct from other auroral phenomena, and allowing visualizing micro-ozone holes. We found ozone destruction of as much as 10–60% within 1.5 h of the initiation of IPA. Electromagnetic ion cyclotron waves in the oxygen ion band observed as the driver of REP likely affect through resonance with mainly ultra-relativistic (> 2 mega-electron-volts) energy electrons. The rapid REP impact demonstrates its crucial role and direct effect on regulating the atmospheric chemical balance.

## Introduction

Atmospheric effects due to energetic particle precipitation (EPP) at hundreds of kilo-electron-volts (keV) to mega-electron-volts (MeV) have significant atmospheric chemical impacts in the mesosphere (50 to 80 km) and the upper stratosphere (~ 50 km) below the thermosphere (100 to 200 km)^1–4^. EPP is one of the major sources of the catalytic destruction of ozone in the polar region (magnetic latitudes > 55°) due to the production of EPP-driven odd nitrogen (NOx) and odd hydrogen (HOx)^5–8^. Plasma particle energy is important for determining the altitude of ionization in the atmosphere^9﻿^. EPP-NOx interactions affect the vertical transport of EPP-driven NOx in the lower thermosphere (its generation altitude of ~ 80 km) down to stratospheric altitude as a so-called indirect effects^10^. As a result, the polar vortex effectively transports NOx in the polar region, then NOx transport plays a significant role in mesospheric ozone loss by 10 to 20% on a time scale of several months to decades^11^. In contrast, the local production of the EPP-driven NOx and HOx directly contributes to ozone destruction at their production altitudes^9,12^. EPP-HOx interactions, particularly, can rapidly occur with a shorter time period of hours, because of the lifetime of a few hours of the HOx family^4^. The ozone density in the mesosphere is much smaller than that in the stratosphere, but the mesospheric ozone and atmospheric ionizations may play an important role for the global climate system via the chemistry and transport processes^13,14^. Solar proton events, which bring a strong enhancement in energetic proton flux (> 10 MeV) caused by an active solar eruption, are a major source of EPP, and such solar proton precipitation plays an important role in ozone destruction^5–7^ and abnormal electron density enhancement in the whole polar cap region^15^. Given that effects from solar proton events are global, we are prompted to ask a new question: can EPP direct impacts on atmospheric chemistry be observed in a localized area with a short duration? In principle, EPP can show clear localization like aurora at specific latitudes, longitudes, and appearance periods. Thus, if EPP directly and rapidly acts as a major driver for the mesospheric ozone destruction as the same with solar proton events, localized ozone destruction associated with localized EPP should be observable. Simulation studies of the direct effect in for specific EPP events (pulsating aurora (> 200-keV electrons)^16^ and relativistic electron microbursts (> 1-MeV electrons)^17^) have predicted up to ~ 20% ozone destruction in the mesosphere, which can be equivalent to the effects created by solar proton events^18^. Such simulation studies predict ozone loss associated with spatially and temporally localized EPP events. However, to date, no observational evidence has verified such localized ozone loss, because identifying any localized, short-duration EPP events in observations is difficult.

In this study, we overcome this difficulty using a specific auroral phenomenon, the so-called isolated proton aurora (IPA) associated with Pc1 geomagnetic pulsations^19^. Most auroras seen at magnetic latitudes usually from 65° to 70° (i.e., in the “auroral oval”) are caused by the particle precipitation of low-energy electrons (keV to tens of keV range), which cannot penetrate to the mesosphere, but the IPA at subauroral latitudes (magnetic latitudes from 55° to 60°), although mostly energized by protons, is accompanied by relativistic electron precipitation (REP) from the outer Earth’s radiation belt^20,21^, which is a torus-shaped zone of relativistic energetic electrons around the Earth’s magnetosphere^22^. The IPA is caused by cyclotron resonant interactions of ring current (tens to a few hundred keV) protons^23,24^ and radiation belt (1- to 10-MeV) electrons^25,26^ with electromagnetic ion cyclotron (EMIC) waves^27–29^, which are coherent electromagnetic wave emissions in the Earth’s magnetosphere and are observed as Pc1 geomagnetic pulsations of frequency a few Hz on the ground. The precipitating protons cause the IPA to emit with characteristic hydrogen optical emissions at subauroral latitudes, making it a proxy for the spatial distribution of REP^21^. The clear temporal correlation between IPA and EMIC/Pc1 waves is further useful as a proxy for the temporal localization of REP^30,31^. Our results demonstrate that EMIC-driven IPA visualizes the localization of the mesospheric ozone loss, like a “needle hole” in the ozone layer, due to precipitation of the Earth’s radiation belt electrons. Quantifying the direct effects of EMIC-driven REP on the atmospheric chemistry and confirming that it accounts for ozone accumulation in such a needle hole contributes to a better understanding of the Sun-magnetosphere-climate connection.

## Results

### Simultaneous IPA and ozone loss observations

**Figure 1:**
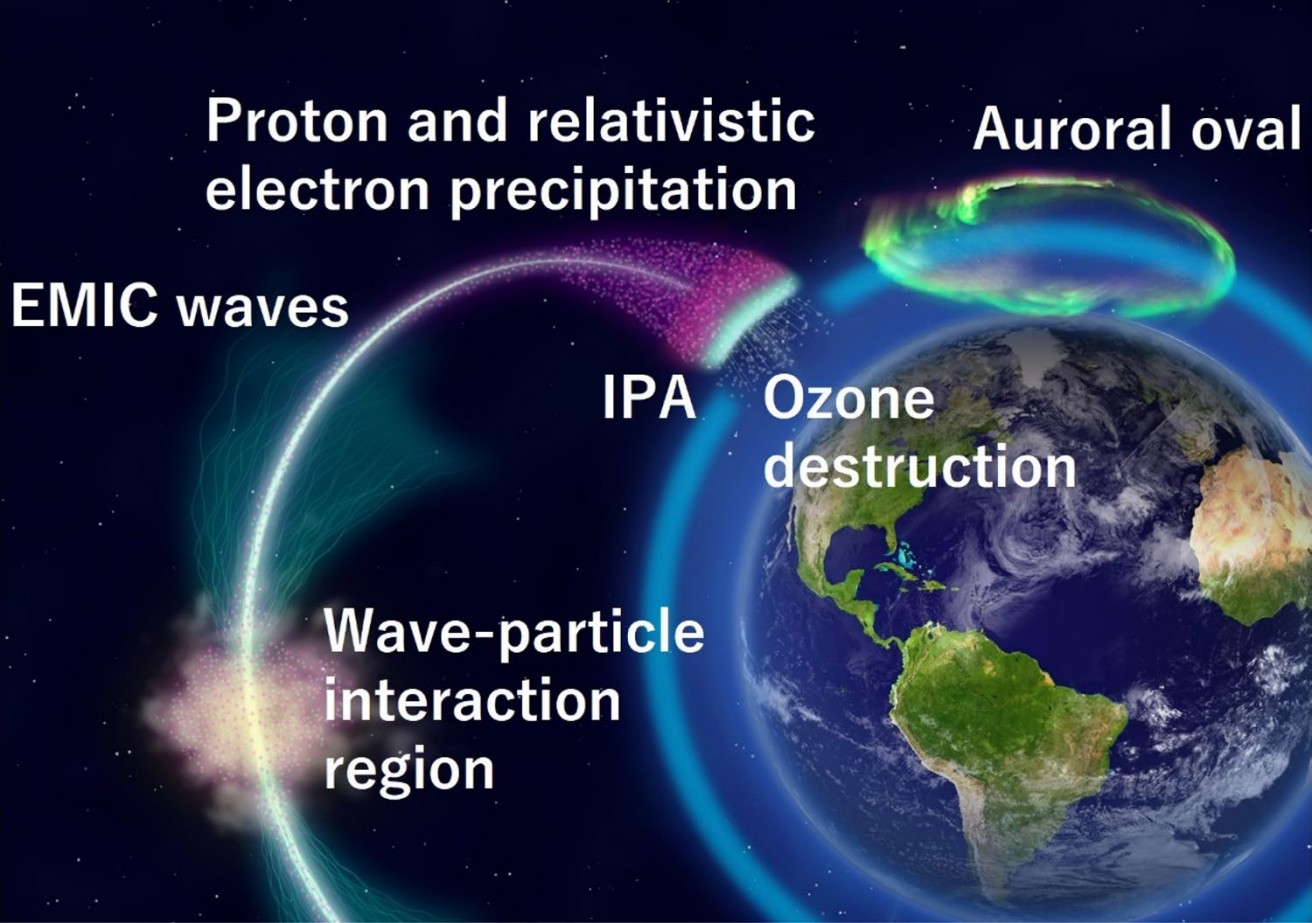
Effects of EMIC wave-particle interactions at different altitudes. Both EMIC-driven protons (about tens of kilo-electron-volts) and relativistic electrons precipitate into the upper atmosphere along the same geomagnetic field lines from the magnetosphere. The localized IPA optical emission due to the protons and the mesospheric ozone destruction by relativistic electron precipitation (REP) can be seen on the same field line at different altitudes. The Earth image is obtained from NASA, https://earthobservatory.nasa.gov/features/BlueMarble/BlueMarble_2002.php. This illustration created with Adobe Illustrator version 26.2.1 (https://www.adobe.com/).

Figure 1 shows a schematic illustration of localized mesospheric ozone loss associated with an IPA. The IPA optical emission serves as an ionospheric indicator of the concentrated downpour of REP. The MeV electrons and lower energy ring current protons are scattered through EMIC wave-particle interactions at the magnetic equator^32^ so that some of them precipitate following their geomagnetic field lines; then the IPA from the protons and accompanying ozone loss due to the MeV electron precipitation (i.e., REP) can be seen at the same location at different altitudes. We utilize innovative satellite remote sensing for identifying IPA and ozone profiles (see “Methods”). The satellite remote sensing of IPA and ozone can allow large spatial coverage regardless of surface weather conditions. The IPA at subauroral latitudes is separated from the equatorward boundary of the auroral oval. In satellite-based limb observations of ozone, this separation is enough to distinguish IPA effects from those of other auroral phenomena near an ozone observation tangent point, which is a point where the line of sight of a sounding ray is closest to the Earth’s surface. The related EMIC waves in the magnetosphere propagate to the ground along the magnetic field line. The EMIC/Pc1 waves are observed at magnetic conjugate ground stations in both the northern and southern hemispheres^33^. The temporal information of IPA is supplemented by a ground observation of related EMIC/Pc1 waves at a fixed ground station. Here we show two prime examples of localized ozone loss related to the IPA.

**Figure 2:**
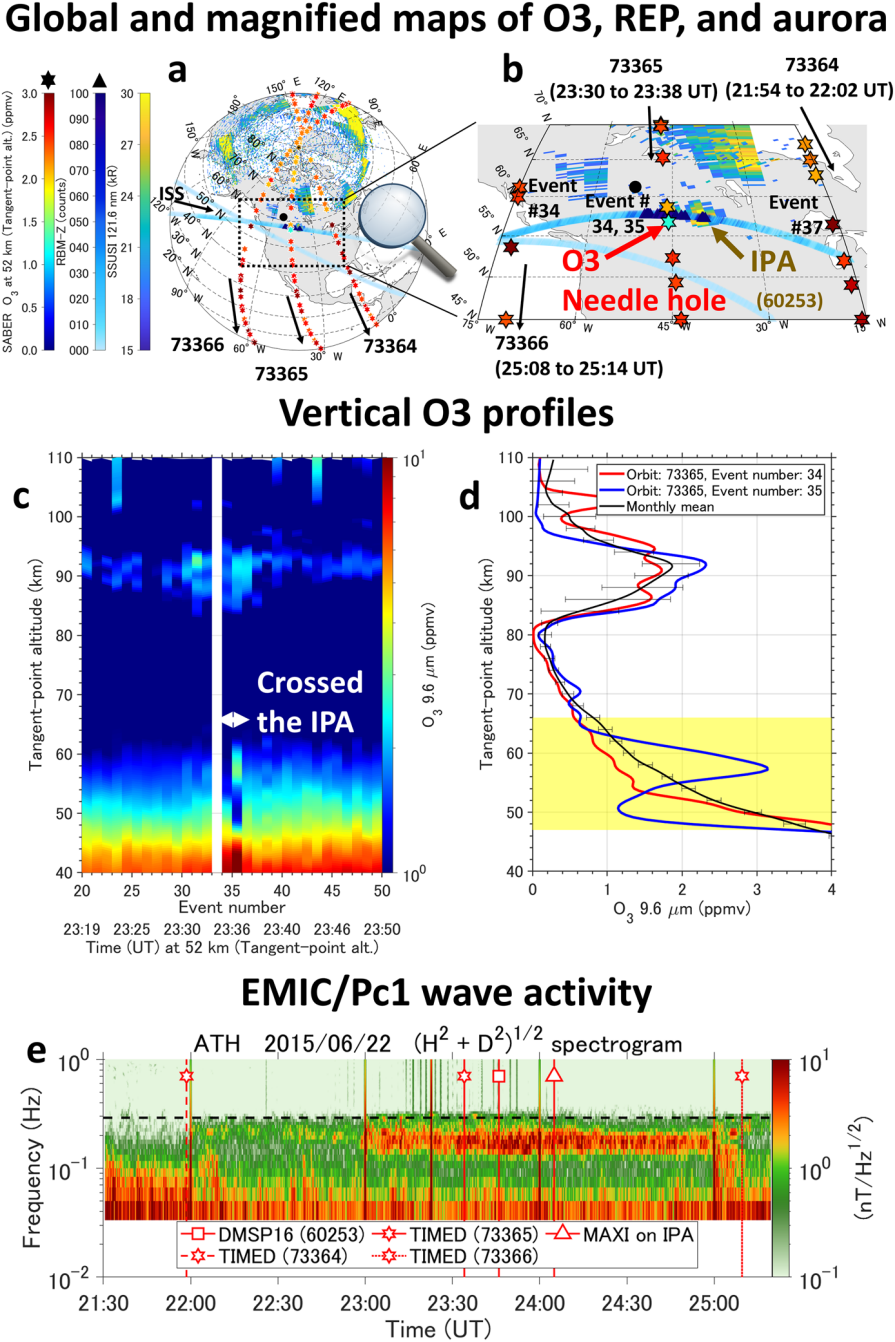
Localized ozone loss associated with the EMIC-driven IPA during a geomagnetic storm. (**a**) Global map of the mesospheric ozone (SABER, stars), REP (RMB-Z, triangles $\ge 90$ counts), and aurora (superposition acquired from 19:44 to 25:46 UT by DMSP18 and 16/SSUSI). (**b**) Magnified map (aurora taken at 23:45 UT by DMSP16/SSUSI). Black arrows indicate the orbit direction of the ISS and TIMED satellite, with the orbit number and the time information in parentheses giving the measurement time for TIMED/SABER. The black dot indicates the location of Athabasca (ATH), Canada. Maps were generated by MATLAB R2020b, https://www.mathworks.com. (**c**) Tangent altitude profile of ozone during orbit 73,365 through the IPA. (**d**) Difference in the mesospheric ozone profiles. “Needle hole” region highlighted. (**e**) EMIC/Pc1 wave activity on June 22, 2015, at ATH. The black dotted line is the O + gyrofrequency at the magnetic equator estimated from an empirical geomagnetic field model^38^. Red lines indicate the time each instrument passed the magnetic latitude of the IPA (~ 57°). Numbers in parentheses denote the orbit number of each satellite.

Figure 2a and b show a coordinated observation of IPA [Lyman alpha (121.6 nm) hydrogen emissions] by the Special Sensor Ultraviolet Spectrographic Imager (SSUSI)^34^ on board the Defense Meteorological Satellite Program (DMSP) satellites and the mesospheric (altitude of 52 km) ozone profiles (stars) by the Sounding of the Atmosphere using Broadband Emission Radiometry (SABER)^35^ on board the Thermosphere Ionosphere Mesosphere Energetics Dynamics (TIMED) satellite from 21:30 to 25:20 UT (13 to 17 magnetic local time (MLT) in the dusk sector), June 22, 2015, during the early main phase of a large geomagnetic storm (minimum Dst index =  − 121 nT). A localized REP is seen, as opposed to the effects of solar proton precipitation, which would be observed in the whole polar cap region^15^. The related REP (triangles) is measured by the vertical component of the Radiation Belt Monitor (RBM-Z) of the Monitor of All-sky X-ray Image (MAXI)^36^ on board the International Space Station (ISS). The global image is plotted in dipole coordinates using the coefficients from the 13th International Geomagnetic Reference Field model^37^. The coordinated IPA and the ozone observation shows a spatial correspondence between the localized IPA and the ozone needle hole. The IPA is seen at a magnetic latitude of ~ 57°, far from the auroral oval boundary at around 62° north latitude. The spatial scale of the IPA is 390 km in the latitudinal direction and 1340 km in the longitudinal direction. The transverse size across the geomagnetic field line of the EMIC wave-particle interaction region is 0.3 to 0.5 Earth radii (Re) in the radial direction, as determined by mapping to the magnetic equator using an empirical geomagnetic field model^38^. The IPA and REP distributions at the magnetic equator were projected from the L = 4.3 to 4.8, MLT = 16 to 17 region. RBM-Z also shows a perfect spatial agreement between the REP and IPA in the longitudinal direction, reflecting the increasing count rate coming from measuring loss cone electrons as REP. Figure 2c shows the altitude profile of ozone measured by the SABER along tangent points of orbit 73,365 of the TIMED satellite just crossing the IPA location. Figure 2d shows the ozone concentration difference between the ozone profiles crossing the IPA and the monthly mean value, with the standard deviation. The monthly mean near the IPA location is calculated from the SABER observations in magnetic latitudes from 55°N to 60°N and longitudes from 15°W to 60°W during 22 to 25 UT between May 23 and June 21, 2015. A significant ozone loss during orbit 73,365 crossing the IPA location during event numbers 34 and 35 is identified at the altitude from 47 to 66 km in the upper stratosphere. A positive bump of ozone around an altitude of 58 km during event number 35 (blue curve) would be caused by the effects of vertical wind shear due to the localized REP^39^. The average ozone loss at the altitude from 47 to 66 km during event numbers 34 and 35 shows a decrease by 11% from the monthly mean value and by 17% from the ozone measurements in the vicinity of the IPA, but outside the IPA in the TIMED orbits 73,364 and 73,366 (see Supplementary Fig. S1). The related EMIC/Pc1 waves in the oxygen ion (O +) band were observed on the ground station at Athabasca (ATH, L = 4.5)^40^, Canada, as shown in Fig. 2e. The EMIC/Pc1 waves were generated at 22:05 UT, and then TIMED/SABER observed the ozone needle hole below the IPA at 23:36 UT during TIMED orbit 73,365. Thus, the ozone variation below the IPA can decrease by up to 17% more in 1.5 h after the generation of EMIC/Pc1 waves with respect to the surrounding region of the IPA.

**Figure 3:**
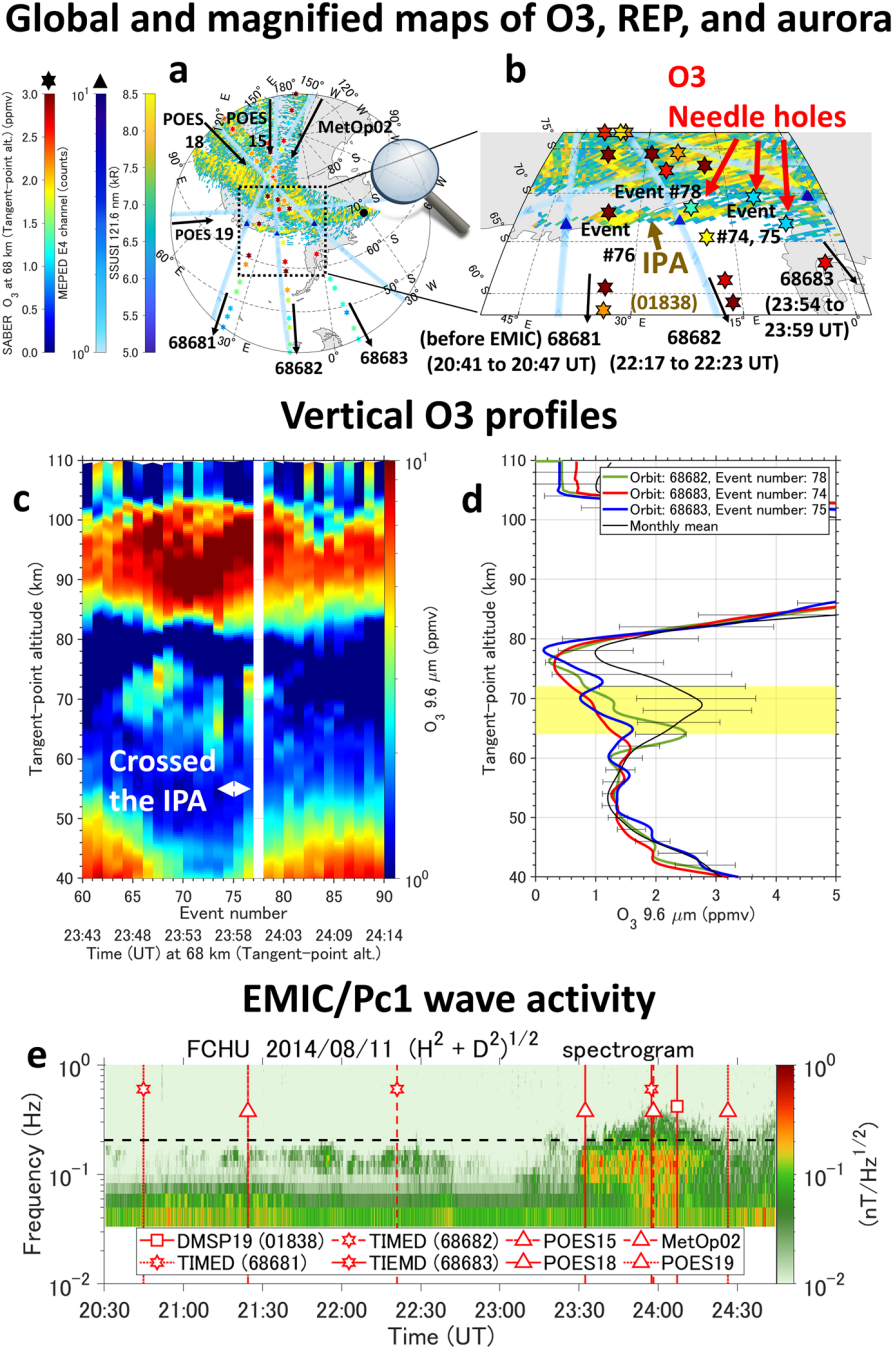
Localized ozone loss associated with EMIC-driven IPA in the absence of a storm. (**a**) Global map of the mesospheric ozone (SABER, stars), REP (MEPED, triangles $\ge 4$ counts), and aurora (superposition acquired from 22:18 to 27:32 UT by DMSP19/SSUSI). The black dot indicates the magnetic conjugate point of Fort Churchill (FCHU), Canada. (**b**) Magnified map (aurora taken at 24:07 UT by DMSP19/SSUSI). Black arrows indicate the orbit direction of the TIMED and POES satellites and the time information in parentheses is the measurement time for TIMED/SABER. Maps were generated by MATLAB R2020b, https://www.mathworks.com. (**c**) Tangent altitude profile of ozone by orbit 68,683 through the IPA. (**d**) Difference in the mesospheric ozone profiles. e EMIC/Pc1 wave activity on August 11, 2014, at FCHU. The black dotted line is the O + gyrofrequency at the magnetic equator estimated from an empirical geomagnetic field model^41^. Red lines indicate the time each instrument passed the same magnetic latitude of the IPA (~ 63°). Numbers in parentheses denote the orbit number of each satellite.

The crucial role of EMIC-driven REP in decreasing mesospheric ozone is further shown in Fig. 3 for a different, non-storm event during 20:30 to 24:45 UT (14 to 18 MLT in the dusk sector), on August 11, 2014. The IPA arc was distributed with a narrow latitude range of 360 km and a wide longitudinal width of 70° (Fig. 3a and b). The equatorial transverse size of the EMIC wave-particle interaction region is 0.8 to 1.0 Re in the radial direction using an empirical geomagnetic field model^41^. The IPA and REP distributions at the magnetic equator were projected from the L = 4.7 to 5.7, MLT = 20 to 22 region. Figure 3 has the same format as Fig. 2, but the REP was measured with the E4 channel of the Medium Energy Proton/Electron Detector (MEPED)^42^ aboard the Polar-orbiting Operational Environmental Satellite (POES) constellation, and the related EMIC/Pc1 waves in the O + band (Fig. 3e) were observed on the ground station at Fort Churchill (FCHU, L = 7.2)^43^, Canada, which is a geomagnetic conjugate point for the IPA observed in Antarctica. After weak EMIC/Pc1 waves were generated at 21:15 UT, POES 15 observed the related REP with a 40-degree wide longitudinal range at 21:24 UT, POES 18 at 23:32 UT, and MetOP02 at 23:57 UT. TIMED/SABER observed the ozone loss during orbits 68,682 and 68,683, which crossed the IPA at different longitudes. A dense mesospheric ozone cloud is seen around altitudes from 62 to 75 km, but a clear destruction of the ozone cloud is seen at event numbers 74 and 75 during the TIMED/SABER orbit 68,683, when it crossed the IPA region (Fig. 3c). In Fig. 3d, the monthly mean of ozone near the IPA location is calculated from the SABER observations at magnetic latitudes from 65**°**S to 70**°**S and longitudes from 0**°**E to 45**°**E during 21:30 to 24:30 UT between July 11 and August 9, 2014. The ozone loss at altitudes from 64 to 72 km observed during event numbers 74, 75, and 78 of TIMED/SABER orbits 68,683 and 68,682 decreased by 51% from the monthly mean value. The ozone loss in event numbers 74 and 75 of TIMED/SABER orbit 68,683 was 61% from the ozone measurements by event numbers 73 and 76 of the same orbit 68,683, but they are observed outside the IPA (see Supplementary Fig. S2). The ozone variation below the IPA is observed to more rapidly decrease ~ 1 h after the generation of EMIC/Pc1 waves compared with that in the surrounding region of the IPA. The clear spatial agreement between the IPA and the mesospheric ozone destruction strongly supports the idea that the EMIC-induced REP directly and rapidly affects the ozone decreases via direct ionization in the mesosphere.

### EMIC-driven REP

**Figure 4:**
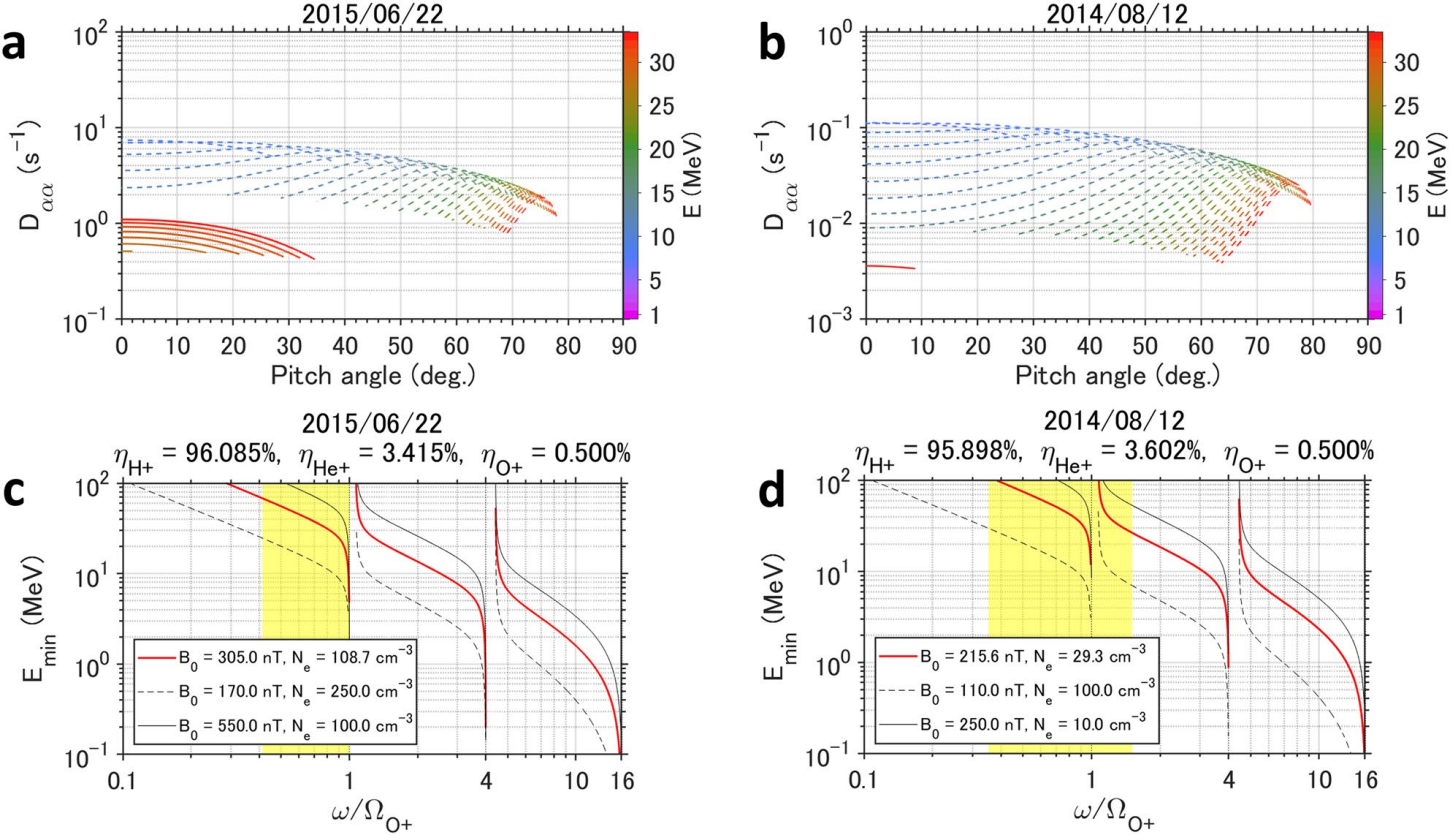
Equatorial pitch angle diffusion and the minimum resonant energy of electrons for observed conditions. (**a**, **b**) Equatorial pitch angle diffusion as a function of equatorial pitch angle. Each curve is plotted every 1 MeV. Solid curves are the calculation results using observed conditions and dotted curves are for a low geomagnetic field strength and a high electron density. (**c**, **d**) Minimum resonant energy as a function of the EMIC wave frequency normalized by the cyclotron frequency of O + ions ${\Omega }_{\mathrm{O}+}$. The yellow rectangle indicates the observed frequency range of the EMIC/Pc1 waves, where ${B}_{0}$ is the geomagnetic field intensity at the magnetic equator and ${N}_{e}$ is the electron density. Red bold curves are the calculation results using observed conditions. Black solid and dotted curves are for the typical values of ${B}_{0}$ and ${N}_{e}$.

The frequency bands of EMIC/Pc1 waves are characterized by related ion species (e.g., proton (H +), Helium (He +), and Oxygen (O +) etc.), and the frequency band is important for determining the resonance plasma energy^25^. EMIC/Pc1 waves along with the IPA are usually observed in the helium ion (He +) band^20,21,30,31^, but EMIC/Pc1 waves in both events studied were in the O + band. We evaluated the equatorial pitch angle diffusion rates^25^ (see “Methods”) and the minimum resonant energy of electrons by the observed EMIC/Pc1 waves to confirm the validation of the EMIC-driven REP. The minimum resonant energy $E_{{{\text{min}}}}$ of electrons interacting with EMIC waves is written as^25^

$$E_{{{\text{min}}}} = \left( {\sqrt {\frac{{{\Omega }_{e}^{2} }}{{\omega^{2} n^{2} }} + 1} - 1} \right)E_{0} ,$$

where ${\Omega }_{e}$ is the electron gyrofrequency; $\omega$ is the angular frequency of EMIC waves; *n* is the refractive index of EMIC waves, which depends on the number density of each ion; and $E_{0}$ is the rest mass energy of an electron (0.511 MeV). Figure 4 shows the equatorial pitch angle diffusion rate (panels a and b) and the minimum resonant energy (panels c and d) for an EMIC wave packet. We used the global core plasma model (GCPM)^44^ 2.2 for the electron and ion densities, and empirical geomagnetic field models^38,41^ with the observed date and location. We assumed parallel plane wave propagation of EMIC waves at the equator to simplify the calculation. Oblique propagation of EMIC waves can reduce the efficiency of REP, but the dependence on the wave normal angles is minor in the case of EMIC waves in the O + band^45^, so our assumption using parallel propagation is adequate. The equatorial diffusion rates under the observed conditions are given in an extremely high energy range > 10 MeV, but the diffusion rates in the case of a typical low geomagnetic field strength (170 nT and 110 nT for the 2015 and 2014 events, respectively) and a high cold electron density (250 cm^−3^ and 100 cm^−3^) are given in ultra-relativistic energies > 2 MeV over a wide pitch angle range (see Fig. 4a, b). EMIC waves in the H + and He + bands cannot typically resonate with ultra-relativistic electrons in such a wide pitch angle range at the equator^25^. Such EMIC waves mainly affect the pitch angle scattering of a few MeV electrons^25,46^, but the O + band EMIC waves effectively enhance the loss of ultra-relativistic (> 2 MeV) electrons. The wave frequency^46^ and hot ion density^47,48^ are also quite important for the loss of radiation belt electrons. This essential pitch angle scattering of ultra-relativistic electrons by EMIC waves is similar to that reported in previous studies^49,50^. Furthermore, non-resonant electrons at lower energies of up to ~ 100 keV, which are below the resonance cutoff of MeV energies in Fig. 4c and d, can still precipitate due to non-resonant interactions by EMIC wave packets with narrow edges^51^. The effects of non-resonant electrons are not taken into account the calculated diffusion rates, and such ~ 100 keV electron precipitation can still produce important atmospheric impacts because of a much larger population of ~ 100 keV electrons than that for ultra-relativistic electrons^49,50^.

Since the observed amplitudes of EMIC/Pc1 waves in both events are close to several % of the background geomagnetic field at the equator, the nonlinear pitch angle scattering^26,52,53^ can significantly contribute to the observed REP events. The typical values of the quasi-linear diffusion rates of the radiation belt electrons range from several hours to a day, but the nonlinear pitch angle scattering by large amplitude EMIC waves having rising-tone frequency structures can cause more rapid (< 1 min) precipitation^26^. In the nonlinear effects, the radiation belt electrons having high pitch angles are guided to lower pitch angles by nonlinear wave trapping from the large amplitude EMIC waves^53^, but it is not sufficient to push them into the loss cone. Then, strong radiation belt electron precipitation is caused by a nonlinear scattering process at low pitch angles by the EMIC waves without nonlinear wave trapping^53^. The combination between nonlinear wave trapping and the scattering at low pitch angle by nonlinear effects of large amplitude EMIC waves leads to effective and rapid precipitation of radiation belt electrons into the atmosphere, with a timescale on the order of seconds, like radiation belt electron microbursts^53^. Previous observational studies on the relativistic electron microbursts in a co-located IPA^54^ and on 1-Hz IPA luminous modulations associated with rapid temporal variations of cosmic noise absorption^55^ support the existence of fast REP on the order of seconds by nonlinear effects. Therefore, the nonlinear effects by large amplitude EMIC waves can strongly contribute to the rapid REP events.

## Discussion

In this study, a clear spatial correspondence between mesospheric ozone loss and localized REP from the Earth’s radiation belt was identified using the IPA during both geomagnetic storm and non-storm conditions. The unprecedented clear correspondence between the EMIC-driven IPA location and the localized mesospheric ozone loss was locally shown on a narrow spatial scale of ~ 400 km in the latitudinal direction. The observed IPA shows a wide longitudinal range over 1000 km, with the effects of the mesospheric jet, up to 60 m s^−1^ in mainly an east to west direction^56^, too small to be important for transport of ozone in the studied events. The mesospheric ozone can quickly respond within 1 to 1.5 h after the EMIC-driven REP. Another candidate driver of REP is whistler mode chorus waves^57,58^, but chorus waves cannot scatter the energetic protons causing the IPA. The direct link between IPA and mesospheric ozone destruction is extremely useful for quantitatively understanding of the atmospheric impacts on the loss of the radiation belt electrons by EMIC waves without the effects of chorus waves. Precipitation of MeV electrons was observed by the MAXI and MEPED instruments during both events as a concentrated downpour of electrons, but the observed EMIC/Pc1 waves in the O + band can resonate with ultra-relativistic electrons beyond the observed range of a few mega-electron-volts. The observed REP events are expected to widely range from the energy covered by MAXI/RBM-Z and MEPED to ultra-relativistic energies. Ultra-relativistic electrons directly penetrate to the mesosphere and the upper stratosphere and create chemical changes in this altitude range^9^. On the other hand, the flux of ultra-relativistic electrons over 10 MeV would be extremely small compared to that of relativistic electrons^49,50^. We have two possibilities to interpret the observed mesospheric ozone destruction. One is the direct effect by the ultra-relativistic (> 2 MeV) electron precipitation, which can strongly interact with EMIC/Pc1 waves in the O + band as shown in Fig. 4. The stopping height of the ultra-relativistic electrons is in the stratosphere below the mesosphere^9^, but the ionization rate of the atmosphere up to the stopping altitude is almost the same as that for the relativistic electrons^9^. Thus, the observed mesospheric ozone destruction can be caused by the effects of the ionization by the ultra-relativistic electrons above the stopping height. The other scenario is the direct effect by precipitating non-resonant electrons in the lower relativistic and/or ~ 100 keV energies^51^. These lower energy electrons as compared to the smaller numbers of ultra-relativistic electrons have a lower fractional scattering efficiency with the EMIC/Pc1 waves, but a much larger population. The lower fractional scattering of electrons of lower energy acting on a much larger population can produce a significant atmospheric impact. If the later scenario is the major reason, the rapid ozone destruction events would be observed during EMIC waves in other ion bands, not only for the O + band. On the other hand, a simulation study showed not only an effective REP, but also a nonlinear blocking of REP by large amplitude EMIC waves^59^. The precipitation blocking was effective at limited low pitch angles less than 10 deg. The precipitating flux of REP can be determined by a balance between the precipitation effects guiding particles into the loss cone in a wide pitch angle range and the precipitation blocking at low pitch angles. Our study should motivate future studies using combined wave and ozone data with incorporation with these precipitation and precipitation blocking effects through wave-particle interaction (quasi-linear, nonlinear, and non-resonance etc.) processes. Identifying the most important EMIC-driven precipitating electron energy to have the major atmospheric impacts remains an open question, so multi-coupled magnetosphere-ionosphere-atmosphere simulations using the quantitative flux measurements of radiation belt electrons into the IPA are also suggested so that further modeling and simulations can investigate the conditions under which immediate ozone destruction processes are created by various wave-particle interaction processes and other atmospheric effects. A previous simulation study suggested a weak mesospheric ozone destruction up to ~ 10% caused by EMIC-driven REP^60^, while the present observations show a greater 10 to 60% ozone destruction, which could have an impact similar to that from other EPP phenomena (pulsating aurora^16^ and microbursts^17^). The accumulated impacts of needle holes in the ozone layer by the IPA cannot be ignored when considering overall ozone changes in the mesosphere.

The EMIC wave-particle interactions shed light on the direct chemical effects of relativistic electrons (REP) on the Earth’s atmospheric processes as an additional EPP source, along with other similar phenomena (e.g., solar proton events, pulsating aurora, and microbursts). Further study is needed to estimate the global effects of the EMIC-driven IPA from individual localized events. The type of IPA events shown here can be excited in different local time sectors at subauroral latitudes^61^. Unfortunately, satellite observations are limited due to orbit and viewing method. It remains unclear how the ozone loss by the IPA occurs in different local time sectors. If the continuous global distribution of IPAs is captured by a ground-based network (such as PWING (study of dynamical variation of Particles and Waves in the INner magnetosphere using Ground-based network observations)^40^) and satellite constellation^62^ observations covering a whole longitudinal range at subauroral latitudes, then quantifying the localized processes of REP-driven chemical impacts in the mesosphere could contribute to an improvement of global circulation models for the middle atmosphere^63,64^. EMIC waves in Jupiter’s magnetosphere similarly scatter energetic heavy ions (sulfur and oxygen) to its ionosphere in a similar manner to the case for Earth, causing similar IPA emissions in the Jovian polar regions^65^. The visualization (“when” and “where”) of the EMIC-driven EPP as IPA provides key insights for understanding the dynamical space weather effects on the atmospheres not only for Earth, but also for the Jovian planets.

## Methods

### Ozone measurements

We used version Level 2A data of ozone from a 9.6 µm wavelength measurement by SABER^35^ on board the TIMED satellite orbiting at an altitude of 625 km with an orbital inclination of 74.1°. The Ozone measurement provides 35% precision in the mesosphere and the upper stratosphere^66^. The advantages of limb sounding by SABER are a precise vertical resolution and a stable background, unlike the variable surface of the Earth. The effect of the low horizontal resolution is not a determinant to this study, because the IPA is seen over a wide (> 1000 km) longitudinal width in a narrow (hundreds of kilometers) latitude.

### Auroral images

To visualize the ionospheric footprint of REP from the Earth’s radiation belt, we used IPA events observed by the SSUSI^34^ aboard the DMSP satellites in an 840-km polar orbit. The Lyman α (121.6 nm) emission by SSUSI is enhanced in the region of the IPA. The signature of the Lyman α emission using a space-based imager that is unaffected by lower atmosphere emissions contributes to identifying the localization of REP with a wide field of view. The auroral image is projected to an altitude of 110 km. The advantage of space-borne observations of auroral images is that the spatial coverage is wide, and observations are unaffected by weather conditions. The ultraviolet wavelength used is also visible when lighting conditions for optical emissions are not favorable. The duration of the IPA and REP is determined from the duration of the related EMIC/Pc1 waves.

### REP measurements

To confirm REP, we used data from the RBM of the MAXI^36^ aboard the ISS orbiting at an altitude of ~ 400 km with an orbital inclination of 51.6°. The MAXI/RBM consists of two sets of PIN diode detectors with horizontal (H) and zenith (Z) directions. The RBM-H and RBM-Z detectors are sensitive to relativistic electrons above 0.3 MeV. In this study, we used RBM-Z data as a proxy for REP into the atmosphere.

The MEPED instrument on board the POES satellite constellation has several energy channels for electrons, but we used the E4 channel data (300 keV–2.5 MeV) from the 0° detector as a typical proxy for the REP^42^.

### Ground-based magnetometers

We focused on EMIC/Pc1 geomagnetic pulsations detected by ground-based magnetometers for identifying EMIC waves in the magnetosphere. The stations are located at Athabasca (ATH, 54.7°N, 246.7°E, L = 4.5)^40^ and Fort Churchill (FCHU, 58.8°N, 265.9°E, L = 7.2)^43^ in Canada. Sampling frequencies of induction magnetometers at ATH and FCHU are 64 Hz and 20 Hz, respectively. The Pc1 wave intensity was calculated from $\sqrt {H^{2} + D^{2} }$, where *H* is the magnetic north–south component and *D* is the east–west component. The short time Fourier transform was used for a 60-s waveform with an overlap of 95%.

### Calculation of pitch angle diffusion rate

When a Gaussian wave spectral density is assumed for an EMIC wave, the pitch angle diffusion rate of relativistic electrons by the EMIC wave is approximately written as^25^

1 $$D_{\alpha \alpha } \approx \frac{{\left| {{\Omega }_{e} } \right|}}{E + 1} \cdot \frac{2R}{{\nu \delta X}} \cdot \frac{{\alpha^{*} \varepsilon X^{2} - Y}}{{2\alpha^{*} \varepsilon X - Z}} \cdot e^{{ - \left( {\frac{{X - X_{m} }}{\delta X}} \right)^{2} }} ,$$

where

$$\begin{aligned} X & = \frac{\omega }{{{\Omega }_{H} }}, \\ Y & = X\left( {\frac{1}{1 + \varepsilon X} + \frac{{\eta_{H + } }}{X - 1} + \frac{{\eta_{He + } }}{4X - 1} + \frac{{\eta_{O + } }}{16X - 1}} \right), \\ Z & = \frac{1}{{\left( {1 + \varepsilon X} \right)^{2} }} - \frac{{\eta_{H + } }}{{\left( {X - 1} \right)^{2} }} - \frac{{\eta_{He + } }}{{\left( {4X - 1} \right)^{2} }} - \frac{{\eta_{O + } }}{{\left( {16X - 1} \right)^{2} }}, \\ \end{aligned}$$

${\Omega }_{e}$ is the electron cyclotron frequency, $E = \frac{{E_{k} }}{{m_{e} c^{2} }}$ is the dimensionless electron kinetic energy of the electron energy $E_{k}$, $m_{e}$ is the electron rest mass, $c$ is the speed of light, $R$ is the ratio of the magnetic field wave power density to the background magnetic field power density, $\nu = \sqrt \pi\cdot {\text{erf}}\left( 1 \right) \approx 1.49$, ${\text{erf}}$ is the error function, $\alpha^{*} = \frac{{{\Omega }_{e}^{2} }}{{\omega_{pe}^{2} }}$, $\omega_{pe}$ is the electron plasma frequency, and $\varepsilon = \frac{{m_{e} }}{{m_{p} }}$ is the mass ratio between the electron rest mass $m_{e}$ and the proton rest mass $m_{p}$. The exponential function in Eq. (1) is derived from a Gaussian wave spectral density with

$$X_{m} = \frac{{\omega_{m} }}{{{\Omega }_{H + } }}\;{\text{and}}\;\delta X = \frac{\delta \omega }{{{\Omega }_{H + } }},$$

where ${\Omega }_{H + }$ is the proton cyclotron frequency, $\omega_{m}$ is the center frequency of an EMIC wave, and $\delta \omega$ is the bandwidth of an EMIC wave. We define the density ratio of each ion as $\eta_{H + } = \frac{{n_{H + } }}{{n_{e} }}$, $\eta_{He + } = \frac{{n_{He + } }}{{n_{e} }}$, and $\eta_{O + } = \frac{{n_{O + } }}{{n_{e} }}$, where $n_{e}$, $n_{H + }$, $n_{He + }$, and $n_{O + }$ are the number densities of the cold electrons, protons, He + ions, and O + ions, respectively. We used the magnetic field wave power density of EMIC waves of 10 nT for a Gaussian spectrum with $\omega_{m} = 0.1875$ Hz and $\delta \omega = 0.0625$ Hz for the June 22, 2015, event, and the magnetic field wave power density of 1 nT with $\omega_{m} = 0.1275$ Hz and $\delta \omega = 0.0575$ Hz for the August 12, 2014, event in Fig. 4. The wave normal angle was simply assumed to be that of parallel plane wave propagation with respect to the background geomagnetic field line.

## Supplementary Information

Supplementary Information.

## Data Availability

TIMED SABER data are available from the website http://saber.gats-inc.com/data.php. DMSP SSUSI data used in this study are publicly available from https://ssusi.jhuapl.edu/data_products. MAXI RBM data were obtained from https://data.darts.isas.jaxa.jp/pub/maxi/rbm/. POES MEPED data were obtained from https://www.ngdc.noaa.gov/stp/satellite/poes/dataaccess.html. EMIC/Pc1 wave data at ATH and FCHU were obtained from https://stdb2.isee.nagoya-u.ac.jp/magne/ and https://www.carisma.ca/, respectively. The Dst index used in this study was provided by the WDC for Geomagnetism, Kyoto (http://wdc.kugi.kyoto-u.ac.jp/wdc/Sec3.html, https://isds-datadoi.nict.go.jp/wds/10.17593__14515-74000.html).

## Code availability

The geomagnetic map was drawn using the mapping package M_Map for MATLAB, available online at www.eoas.ubc.ca/~rich/map.html.

## References

[1] Rodger CJ, Clilverd MA, Thomson NR, Gamble RJ, Seppälä A, Turunen E, Meredith NP, Parrot M, Sauvaud J-A, Berthelier J-J (2007). Radiation belt electron precipitation into the atmosphere: recovery from a geomagnetic storm. J. Geophys. Res..

[2] Rozanov E, Calisto M, Egorova T, Peter T, Schmutz W (2012). Influence of the precipitating energetic particles on atmospheric chemistry and climate. Surv. Geophys..

[3] Seppälä A, Matthes K, Randall CE, Mironova IA (2014). What is the solar influence on climate? Overview of activities during CAWSES-II. Prog. Earth Planet. Sci..

[4] Andersson M, Verronen P, Rodger C (2014). Missing driver in the Sun-Earth connection from energetic electron precipitation impacts mesospheric ozone. Nat Commun..

[5] Heath DF, Kruger AJ, Crutzen PJ (1977). Solar proton events: influence on stratospheric ozone. Science.

[6] Jackman CH (2001). Northern hemisphere atmospheric effects due to the July 2000 solar proton events. Geophys. Res. Lett..

[7] Funke B, Baumgaertner A, Calisto M, Egorova T, Jackman CH, Kieser J (2011). Composition changes after the “Halloween” solar proton event: the high-energy particle precipitation in the atmosphere (HEPPA) model versus MIPAS data intercomparison study. Atmos. Chem. Phys..

[8] Solomon S, Rusch DW, Gerard J-C, Reid GC, Crutzen PJ (1981). The effect of particle precipitation events on the neutral and ion chemistry of the middle atmosphere. II—odd hydrogen. Planet. Space Sci..

[9] Turunen (2009). Impact of different energies of precipitating particles on NOx generation in the middle and upper atmosphere during geomagnetic storms. J. Atmos. Solar Terr. Phys..

[10] Randall CE, Harvey VL, Singleton CS, Bernath PF, Boone CD, Kozyra JU (2006). Enhanced NOx in 2006 linked to strong upper stratospheric Arctic vortex. Geophys. Res. Lett..

[11] Andersson ME (2018). Polar ozone response to energetic particle precipitation over decadal time scales: the role of medium-energy electrons. J. Geophys. Res.: Atmos..

[12] Jia J, Kero A, Kalakoski N, Szeląg ME, Verronen PT (2020). Is there a direct solar proton impact on lower-stratospheric ozone?. Atmos. Chem. Phys..

[13] Baumgaertner AJG, Seppälä A, Jöckel P, Clilverd MA (2011). Geomagnetic activity related NOx enhancements and polar surface air temperature variability in a chemistry climate model: modulation of the NAM index. Atmos. Chem. Phys..

[14] Seppälä A, Lu H, Clilverd MA, Rodger CJ (2013). Geomagnetic activity signatures in wintertime stratosphere wind, temperature, and wave response. J. Geophys. Res. Atmos..

[15] Bornebusch JP, Wissing JM, Kallenrode M-B (2010). Solar particle precipitation into the polar atmosphere and their dependence on hemisphere and local time. Adv. Space Res..

[16] Turunen E, Kero A, Verronen PT, Miyoshi Y, Oyama S-I, Saito S (2016). Mesospheric ozone destruction by high-energy electron precipitation associated with pulsating aurora. J. Geophys. Res. Atmos..

[17] Seppälä A, Douma E, Rodger CJ, Verronen PT, Clilverd MA, Bortnik J (2018). Relativistic electron microburst events: modeling the atmospheric impact. Geophys. Res. Lett..

[18] Verronen PT, Seppälä A, Clilverd MA, Rodger CJ, Kyrölä E, Enell C.-F, Ulich T, Turunen E (2005). Diurnal variation of ozone depletion during the October–November 2003 solar proton events. J. Geophys. Res.

[19] Yahnin AG, Yahnina TA, Frey HU (2007). Subauroral proton spots visualize the Pc1 source. J. Geophys. Res..

[20] Miyoshi Y, Sakaguchi K, Shiokawa K, Evans D, Albert J, Connors M, Jordanova V (2008). Precipitation of radiation belt electrons by EMIC waves, observed from ground and space. Geophys. Res. Lett..

[21] Sakaguchi K, Miyoshi Y, Spanswick E, Donovan E, Mann IR, Jordanova V, Shiokawa K, Connors M, Green JC (2012). Visualization of ion cyclotron wave and particle interactions in the inner magnetosphere via THEMIS-ASI observations. J. Geophys. Res..

[22] Van Allen JA, Frank LA (1959). Radiation around the earth to a radial distance of 107,400 km. Nature.

[23] Usanova ME (2010). Conjugate ground and multisatellite observations of compression-related EMIC Pc1 waves and associated proton precipitation. J. Geophys. Res..

[24] Jordanova VK, Spasojevic M, Thomsen MF (2007). Modeling the electromagnetic ion cyclotron wave-induced formation of detached subauroral proton arcs. J. Geophys. Res..

[25] Summers D, Thorne RM (2003). Relativistic electron pitch-angle scattering by electromagnetic ion cyclotron waves during geomagnetic storms. J. Geophys. Res..

[26] Omura Y, Zhao Q (2013). Relativistic electron microbursts due to nonlinear pitch angle scattering by EMIC triggered emissions. J. Geophys. Res. Space Phys..

[27] Cornwall JM (1965). Cyclotron instabilities and electromagnetic emission in the ultra low frequency and very low frequency ranges. J. Geophys. Res..

[28] Anderson, B. J., Erlandson, R. E., & Zanetti, L. J. A statistical study of Pc 1–2 magnetic pulsations in the equatorial magnetosphere: 1. Equatorial occurrence distributions. *J. Geophys. Res.* **97**(A3), 3075–3088. 10.1029/91JA02706 (1992).

[29] Pickett JS (2010). Cluster observations of EMIC triggered emissions in association with Pc1 waves near Earth's plasmapause. Geophys. Res. Lett..

[30] Nomura R, Shiokawa K, Omura Y, Ebihara Y, Miyoshi Y, Sakaguchi K, Otsuka Y, Connors M (2016). Pulsating proton aurora caused by rising tone Pc1 waves. J. Geophys. Res. Space Phys..

[31] Ozaki M (2016). Fast modulations of pulsating proton aurora related to subpacket structures of Pc1 geomagnetic pulsations at subauroral latitudes. Geophys. Res. Lett..

[32] Loto'aniu TM, Fraser BJ, Waters CL (2005). Propagation of electromagnetic ion cyclotron wave energy in the magnetosphere. J. Geophys. Res..

[33] Ozaki, M., Shiokawa, K., Horne, R. B., Engebretson, M. J., Lessard, M., Ogawa, Y., et al. Magnetic conjugacy of Pc1 waves and isolated proton precipitation at subauroral latitudes: Importance of ionosphere as intensity modulation region. *Geophys. Res. Lett.*. **48**, e2020GL091384. (2021). 10.1029/2020GL091384

[34] Paxton, L. J., Morrison, D., Zhang, Y., Kil, H., Wolven, B., Ogorzalek, B. S., et al. Validation of remote sensing products produced by the Special Sensor Ultraviolet Scanning Imager (SSUSI): A far UV-imaging spectrograph on DMSP F-16. *Proc. SPIE 4485, Optical Spectroscopic Techniques, Remote Sensing, and Instrumentation for Atmospheric and Space Research IV*, 4485, 338–348. (2002). 10.1117/12.454268

[35] Russell, J. M. III, M. G. Mlynczak, L. L. Gordley, J. Tansock, and R. Esplin. An overview of the SABER experiment and preliminary calibration results. *Proc. SPIE* **3756**, 277–288. 10.1117/12.366382 (1999).

[36] Matsuoka M (2009). The MAXI Mission on the ISS: science and instruments for monitoring all-sky X-ray images. Publ. Astron. Soc. Jpn..

[37] Alken P, Thébault E, Beggan CD (2021). International geomagnetic reference field: the thirteenth generation. Earth Planets Space..

[38] Tsyganenko NA (2002). A model of the magnetosphere with a dawn-dusk asymmetry, 1 Mathematical structure. J. Geophys. Res..

[39] Oyama S-I, Shiokawa K, Miyoshi Y, Hosokawa K, Watkins BJ, Kurihara J, Tsuda TT, Fallen CT (2016). Lower thermospheric wind variations in auroral patches during the substorm recovery phase. J. Geophys. Res. Space Physics.

[40] Shiokawa K, Katoh Y, Hamaguchi Y (2017). Ground-based instruments of the PWING project to investigate dynamics of the inner magnetosphere at subauroral latitudes as a part of the ERG-ground coordinated observation network. Earth Planets Space..

[41] Tsyganenko NA, Sitnov MI (2005). Modeling the dynamics of the inner magnetosphere during strong geomagnetic storms. J. Geophys. Res..

[42] Peck ED, Randall CE, Green JC, Rodriguez JV, Rodger CJ (2015). POES MEPED differential flux retrievals and electron channel contamination correction. J. Geophys. Res. Space Physics..

[43] Mann IR, Milling DK, Rae IJ (2008). The upgraded CARISMA magnetometer array in the THEMIS era. Space Sci Rev..

[44] Gallagher DL, Craven PD, Comfort RH (2000). Global core plasma model. J. Geophys. Res..

[45] Ni B (2015). Resonant scattering of outer zone relativistic electrons by multiband EMIC waves and resultant electron loss time scales. J. Geophys. Res. Space Phys..

[46] Albert JM (2003). Evaluation of quasi-linear diffusion coefficients for EMIC waves in a multispecies plasma. J. Geophys. Res..

[47] Ni B, Cao X, Shprits YY, Summers D, Gu X, Fu S, Lou Y (2018). Hot plasma effects on the cyclotron-resonant pitch-angle scattering rates of radiation belt electrons due to EMIC waves. Geophys. Res. Lett..

[48] Cao X, Shprits YY, Ni B (2017). Scattering of ultra-relativistic electrons in the Van Allen radiation belts accounting for hot plasma effects. Sci Rep..

[49] Usanova ME (2014). Effect of EMIC waves on relativistic and ultrarelativistic electron populations: ground-based and Van Allen Probes observations. Geophys. Res. Lett..

[50] Shprits Y, Drozdov A, Spasojevic M (2016). Wave-induced loss of ultra-relativistic electrons in the Van Allen radiation belts. Nat Commun.

[51] Chen L, Thorne RM, Bortnik J, Zhang X-J (2016). Nonresonant interactions of electromagnetic ion cyclotron waves with relativistic electrons. J. Geophys. Res. Space Phys..

[52] Kubota Y, Omura Y, Summers D (2015). Relativistic electron precipitation induced by EMIC-triggered emissions in a dipole magnetosphere. J. Geophys. Res. Space Phys..

[53] Kubota Y, Omura Y (2017). Rapid precipitation of radiation belt electrons induced by EMIC rising tone emissions localized in longitude inside and outside the plasmapause. J. Geophys. Res. Space Phys..

[54] Shumko M (2022). Proton aurora and relativistic electron microbursts scattered by electromagnetic ion cyclotron waves. Front. Astron. Space Sci..

[55] Ozaki M (2018). Discovery of 1 Hz range modulation of isolated proton aurora at subauroral latitudes. Geophys. Res. Lett..

[56] Sato K, Watanabe S, Kawatani Y, Tomikawa Y, Miyazaki K, Takahashi M (2009). On the origins of mesospheric gravity waves. Geophys. Res. Lett..

[57] Summers D, Ni B, Meredith NP (2007). Timescales for radiation belt electron acceleration and loss due to resonant wave-particle interactions: 2. Evaluation for VLF chorus, ELF hiss, and electromagnetic ion cyclotron waves. J. Geophys. Res..

[58] Hsieh, Y.-K., Omura, Y., & Kubota, Y. Energetic electron precipitation induced by oblique whistler mode chorus emissions. *J. Geophys. Res.: Space Phys.* **127**, e2021JA029583, (2022). 10.1029/2021JA029583

[59] Bortnik, J., Albert, J. M., Artemyev, A., Li, W., Jun, C.-W., Grach, V. S., & Demekhov, A. G. Amplitude dependence of nonlinear precipitation blocking of relativistic electrons by large amplitude EMIC waves. *Geophys. Res. Lett.* **49**, e2022GL098365 (2022). 10.1029/2022GL098365 10.1029/2022GL098365 PMC9541690 36246783

[60] Hendry, A. T., Seppälä, A., Rodger, C. J., & Clilverd, M. A. Impact of EMIC-wave driven electron precipitation on the radiation belts and the atmosphere. *J. Geophys. Res.: Space Phys*. **126**, e2020JA028671 (2021). 10.1029/2020JA028671

[61] Semenova NV, Yahnin AG, Yahnina TA, Demekhov AG (2019). Properties of localized precipitation of energetic protons equatorward of the isotropic boundary. Geophys. Res. Lett..

[62] Morley SK, Sullivan JP, Henderson MG, Blake JB, Baker DN (2016). The global positioning system constellation as a space weather monitor: comparison of electron measurements with Van Allen Probes data. Space Weather.

[63] Verronen PT, Andersson ME, Marsh DR, Kovács T, Plane JMC (2016). WACCM-D: whole atmosphere community climate model with D-region ion chemistry. J. Adv. Model. Earth Syst..

[64] Danabasoglu, G., Lamarque, J.-F., Bacmeister, J., Bailey, D. A., DuVivier, A. K., Edwards, J., et al. The community earth system model version 2 (CESM2). *J. Adv. Model. Earth Syst*. **12**, e2019MS001916, (2020). 10.1029/2019MS001916

[65] Yao Z (2021). Revealing the source of Jupiter’s x-ray auroral flares. Sci. Adv..

[66] Rong, P. P., Russell, J. M., Mlynczak, M. G., Remsberg, E. E., Marshall, B. T., Gordley, L. L., & López-Puertas, M. Validation of thermosphere ionosphere mesosphere energetics and dynamics/sounding of the atmosphere using broadband emission radiometry (TIMED/SABER) v1.07 ozone at 9.6 μm in altitude range 15–70 km. *J. Geophys. Res*. **114**, D04306. 10.1029/2008JD010073 (2009).

