# Supplementary material for: Localized mesospheric ozone destruction corresponding to isolated proton aurora coming from Earth’s radiation belt
Source: Sci Rep. 2022 Oct 11;12:16300. doi: 10.1038/s41598-022-20548-2 (PMC9553911; doi:10.1038/s41598-022-20548-2)
Supplement: Supplementary file 1 — Supplementary Information. [file 41598_2022_20548_MOESM1_ESM.docx]

**Supplementary Information**

**Localized mesospheric ozone destruction corresponding to isolated proton aurora coming from Earth’s radiation belt**

Mitsunori Ozaki^1^, Kazuo Shiokawa^2^, Ryuho Kataoka^3, 4^, Martin Mlynczak^5^, Larry Paxton^6^, Martin Connors^7, 8^, Satoshi Yagitani^1^, Shion Hashimoto^1^, Yuichi Otsuka^2^, Satoshi Nakahira^9^ & Ian Mann^10^

1 Graduate School of Natural Science and Technology, Kanazawa University, Kanazawa, Japan.

2 Institute for Space-Earth Environmental Research, Nagoya University, Nagoya, Japan.

3 National Institute of Polar Research, Tachikawa, Japan.

4 Department of Polar Science, The Graduate University for Advanced Studies, SOKENDAI, Tachikawa, Japan.

5 NASA Langley Research Center, Hampton, VA, USA.

6 The Johns Hopkins University Applied Physics Laboratory, Laurel, MD, USA.

7 Athabasca University Observatories, Athabasca, AB, Canada.

8 Department of Physics and Astronomy, University of Calgary, Calgary AB, Canada

9 Institute of Space and Astronautical Science, Japan Aerospace Exploration Agency, Sagamihara, Japan.

10 Department of Physics, University of Alberta, Edmonton, AB, Canada.

Corresponding author

Correspondence to Mitsunori Ozaki.

ozaki@is.t.kanazawa-u.ac.jp


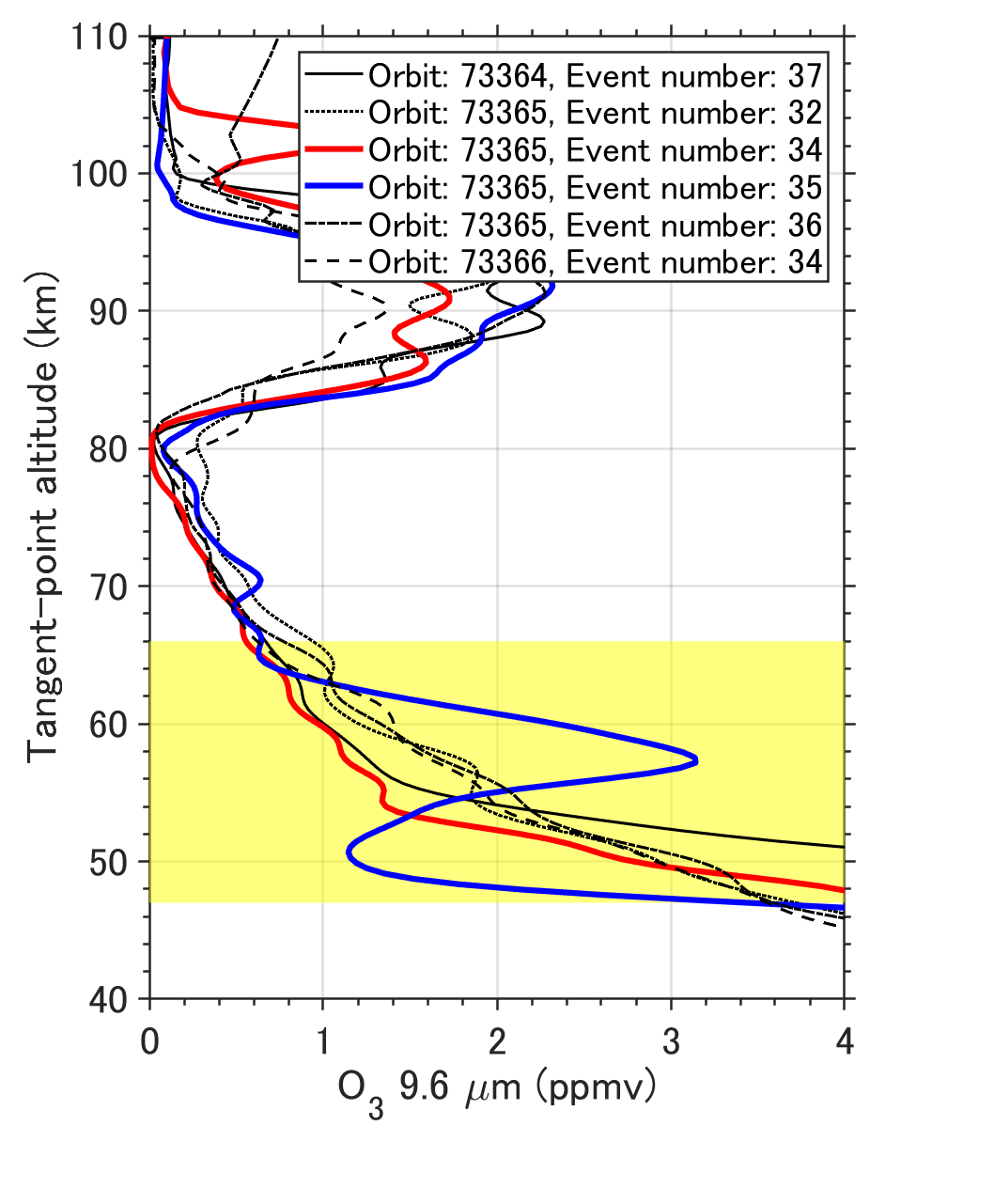


**Supplementary Figure S1. Vertical ozone profiles at locations crossing the IPA (event numbers 34 and 35 of TIMED orbit 73365) and outside of the IPA.**

**Black solid and dotted curves give the ozone concentration outside the IPA. The average ozone loss at altitudes from 47 to 66 km during orbit 73365 (event numbers 34 and 35 crossing the IPA) decreased by 17% from the SABER ozone for the other orbits 73364 (event number 37) and 73366 (event number 34), which did not cross the IPA location. It also decreased by 12% from the SABER ozone in the vicinity of the IPA at event numbers 32 and 36 during the same orbit 73365, which were observed outside the IPA.**


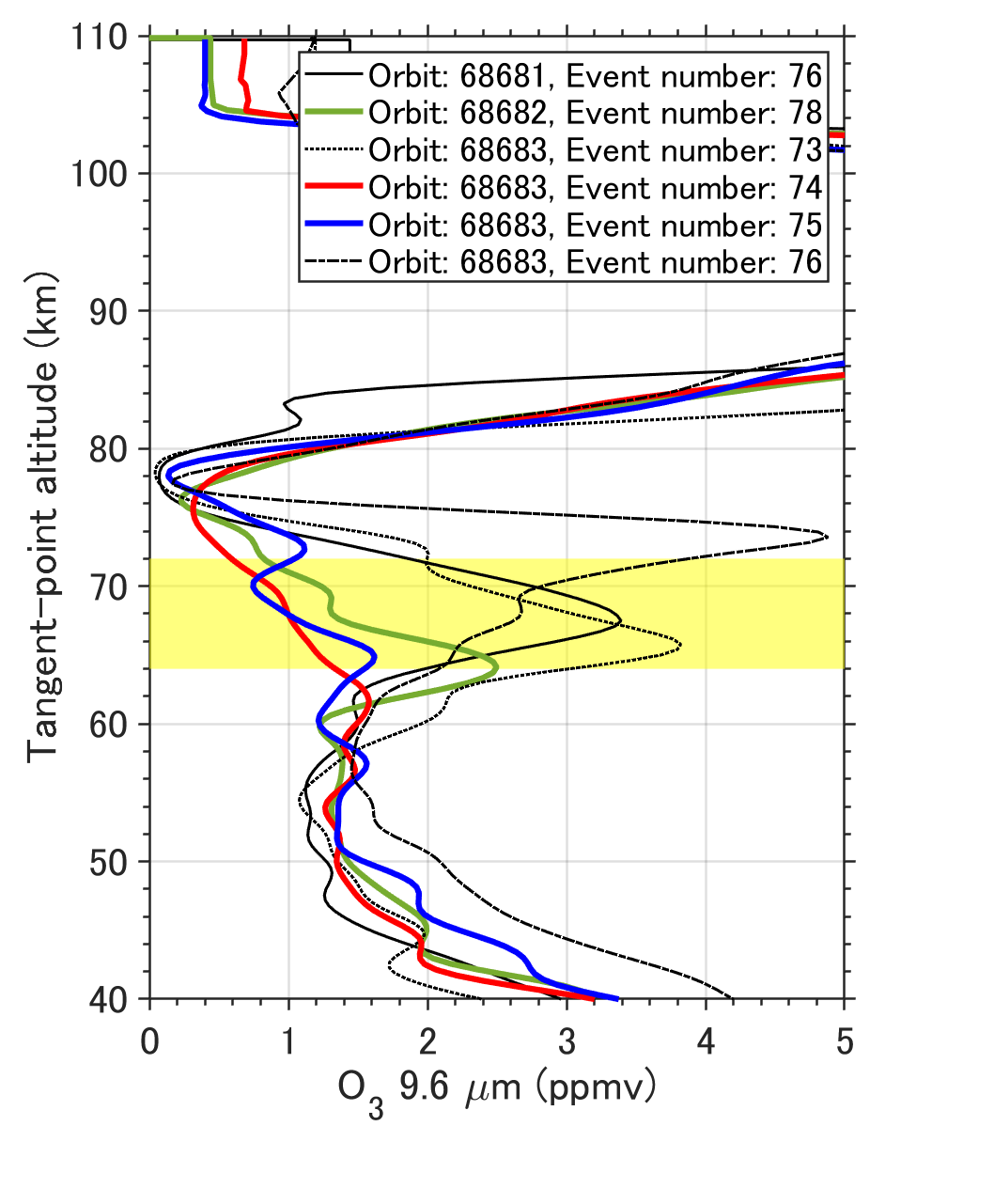


**Supplementary Figure S2. Vertical ozone profiles at locations crossing the IPA (event numbers 74 and 75 of TIMED orbit 68683 and event number 78 of orbit 68682) and outside the IPA.**

**Black solid and dotted curves are outside the IPA. The ozone loss at altitude from 64 to 72 km observed by TIMED/SABER orbit 68683 (event numbers 74 and 75 crossing the IPA) decreased by 40% from that for orbit 68681 (event number 76), which was observed before the generation of the related EMIC Pc1 waves, indicating no REP. The ozone loss from 64 to 72 km seen during TIMED/SABER orbit 68682 (event number 78 crossing the IPA) represented a decrease by 42% from that for orbit 68681 (event number 76). The average ozone loss during orbit 68683 (event numbers 74 and 75) decreased by 61% from the SABER-detected ozone during event numbers 73 and 76, which are for the same orbit 68683, but they were observed outside the IPA.**
